# Mechanical augmentation for vertebral compression fractures: A scoping review

**DOI:** 10.1016/j.inpm.2026.100764

**Published:** 2026-04-19

**Authors:** Hasan Sen, Amelia Y. Ni, Amanda N. Cooper, Gerard Limerick, Alejandra Cardenas-Rojas, Sujeivan Mahendram, Sharima Kanahan Osman, Christopher Radlicz, Jennifer Tram, Masaru Teramoto, Napatpaphan Kanjanapanang, Zachary L. McCormick, Aaron M. Conger

**Affiliations:** a Department of Physical Medicine and Rehabilitation, University of Utah, Salt Lake City, UT, United States; b Department of Physical Medicine and Rehabilitation, Jefferson Health, Philadelphia, PA, United States; c Department of Physical Medicine and Rehabilitation, Johns Hopkins, Baltimore, MD, United States; d Department of Pain Medicine, John Hopkins, Baltimore, MD, United States; e Department of Pain Medicine, Mayo Clinic, Jacksonville, FL, United States; f Division of Physical Medicine and Rehabilitation, Department of Orthopedics, Washington University School of Medicine in St. Louis, St. Louis, MO, United States; g Department of Physical Medicine and Rehabilitation, University of California Los Angeles, Los Angeles, CA, United States

**Keywords:** Vertebral compression fracture, Osteoporosis, Mechanical vertebral augmentation, Kyphoplasty, Cement extravasation

## Abstract

**Background:**

Vertebral compression fractures (VCFs) contribute substantially to pain, disability, and healthcare burden. Vertebroplasty and balloon kyphoplasty (KP) are widely used but limited by cement leakage and inconsistent vertebral height restoration. Mechanical vertebral augmentation (MVA) techniques were developed to improve vertebral alignment and cement containment, yet their comparative outcomes remain incompletely defined.

**Objectives:**

To map and synthesize the existing evidence on MVA compared with KP or vertebroplasty across seven clinically relevant domains, including cement extravasation, alignment correction, pain and disability outcomes, morbidity and mortality, and feasibility in complex fracture patterns.

**Methods:**

A comprehensive search of MEDLINE, Embase, and Web of Science identified studies evaluating MVA in adult VCFs. Data were charted according to seven predefined research questions and synthesized descriptively using scoping-review methodology. Forest plots were used to visualize the ranges and distributions of reported outcomes across studies.

**Results:**

Fifty-two studies met inclusion criteria. Across studies, MVA demonstrated a trend toward lower cement extravasation rates compared with KP, although definitions and detection methods varied and intradiscal leakage was generally reported as asymptomatic. Vertebral height restoration and alignment correction were often greater or more consistently achieved with MVA devices, but the clinical relevance of these differences remains uncertain. However, improved structural correction did not consistently translate into superior clinical outcomes across studies. Adjacent fracture rates ranged widely, with some studies describing lower rates after MVA and others showing similar or higher rates, particularly in cases involving more aggressive correction. Evidence describing morbidity or mortality differences across augmentation techniques was limited, and long-term comparative data remain sparse. Overall, available findings support the feasibility and short-term safety of MVA, though heterogeneity in reporting limits firm conclusions.

**Conclusions:**

Current evidence suggests that MVA is a feasible and safe treatment option for VCFs, including complex morphologies. Although some studies report differences in radiographic correction or cement leakage patterns between MVA and KP, the clinical significance remains uncertain, and improved structural correction does not consistently translate into superior clinical outcomes. High-quality randomized trials with standardized imaging and clinical outcome reporting are needed to clarify how different augmentation strategies compare and whether structural restoration achieved with MVA leads to consistent, meaningful improvements in patient outcomes.

## 1 Introduction

Vertebral compression fractures (VCFs) can lead to pain, disability, reduced quality of life, and decreased pulmonary function due to kyphotic deformity [1,2]. Osteoporotic vertebral compression fractures (OVCFs) are quite prevalent in North America, where a 2017 review found a 20–24% prevalence rate for OVCF among white women over the age of 50 [3]. Subsequent interventional and medical management creates a substantial burden on the healthcare system, with an economic analysis of German claims data from 2005 to 2010 calculating that the yearly mean, unadjusted, all-cause healthcare cost difference between OVCF and OVCF-free patients was €8200 (*p* < 0.001) [4]. Various minimally invasive procedures have been introduced over time that stabilize the fractured vertebral body and alleviate pain: vertebroplasty, kyphoplasty (KP), and most recently, mechanical vertebral augmentation (MVA) [5].

**Fig. 1:**
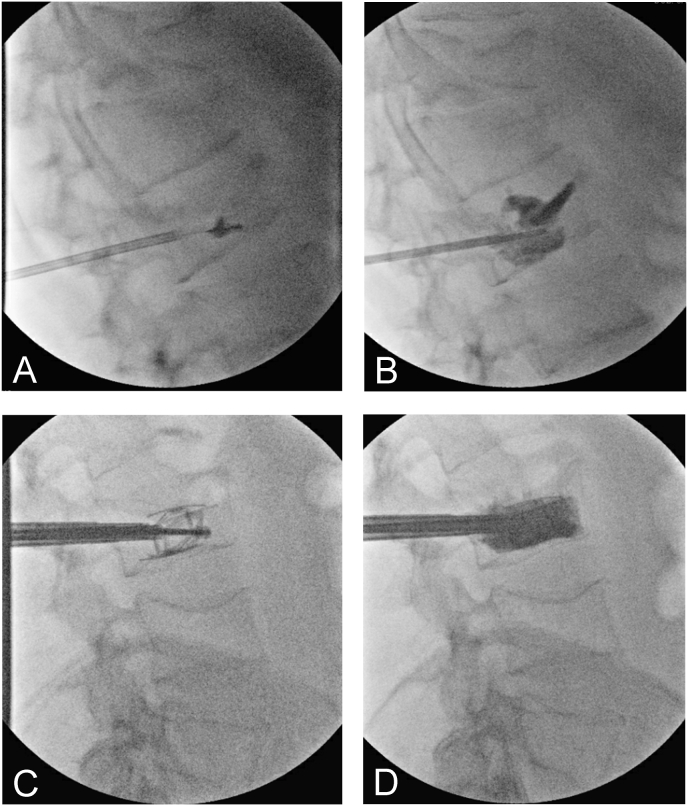
Lateral fluoroscopic images of incomplete burst fractures at L1 (A, B) and L3 (C, D) treated with balloon kyphoplasty and mechanical vertebral augmentation, respectively. Intradiscal cement extravasation is noted at L1.

Vertebroplasty involves the direct injection of cement into the vertebral body, providing rapid pain relief but with increased risk of cement extravasation [6]. KP uses an inflatable balloon to create a void in the vertebral body before cement injection, potentially restoring some height to fractured vertebrae and reducing risk of cement leakage with a more controlled injection (Fig. 1a and b) [7]. MVA is designed to better restore vertebral height while minimizing cement leakage and may lower the risk of adjacent vertebral fracture relative to KP (Fig. 1c and d) [8]. Multiple MVA systems exist, including expandable cages, stent-based devices, and implant-guided systems [6].

Historically the literature has compared vertebroplasty to KP, with the general consensus that, despite the greater cost, KP is safer with less cement extravasation and provides greater height restoration [9,10]. However, comparative evidence between MVA and KP remains limited, particularly regarding cement extravasation and the clinical relevance of disc leakage, overall leakage incidence, the effect of structural alignment on outcomes, the relationship between alignment correction and fracture risk, potential differences in morbidity and mortality, and the feasibility of MVA in severe or complex fractures. Given the breadth of research spanning three generations of vertebral augmentation, we performed a scoping review to determine what evidence exists surrounding these outcomes and developed seven key research questions to define current knowledge and guide future work.

## 2 Methods

The review was guided by seven structured research questions as shown in Table 1, developed using the Population-Concept-Context (PCC) framework [11].

**Table 1:** Research questions structured using the PCC framework.

Research Questions	Population	Concept	Context
RQ1: What are the clinically significant ramifications of cement extravasation into adjacent segment discs during MVA procedures, in isolation or when directly compared to vertebroplasty or KP?	Adults undergoing MVA procedure alone or when compared to either vertebroplasty for the treatment of osteoporotic vertebral compression fractures (OVCF) or traumatic vertebral compression fractures (TVCF), (including and subcategorizing for burst fractures).	Endplate disruption in OVCF and TVCF can lead to cement extravasation into adjacent discs or other undesirable locations, resulting in inadequate fracture stabilization and various complications. Cement within the disc may impair load distribution, increasing the risk of index collapse, adjacent segment fracture, and persistent pain. Key outcomes include adjacent fracture risk, index collapse progression, pain relief, and functional improvement	No geographic, duration of follow up, racial, or gender limitations, all clinical practice settings where fractures are treated with vertebral augmentation.
RQ2: What is the incidence of cement extravasation in MVA procedures in isolation or when directly compared to vertebroplasty or KP?	Adults undergoing MVA procedure alone or when compared to KP for the treatment of OVCF or TVCF	Mechanical augmentation may offer distinct advantages over KP by reducing the risk of cement extravasation and allowing for greater cement fill within the vertebral body. Evaluating the frequency of cement leakage across augmentation techniques is essential for assessing procedural safety	No geographic, duration of follow up, racial, or gender limitations, all clinical practice settings where fractures are treated with vertebral augmentation.
RQ3: What is the overall impact of structural alignment modification from mechanical vertebral augmentation on outcomes in patients with OVCF or TVCF?	Adults undergoing MVA procedures for OVCF or TVCF, as well as cadaveric or digital replica studies examining MVA for these fracture types.	Spinal alignment may influence load distribution and mechanical stress within the spine and significant kyphosis could be associated with increasing anterior column loads, index fracture collapse, adjacent segment fracture risk, continued pain or continued disability in the setting of OVCF or TVCF	No geographic, duration of follow up, racial, or gender limitations, all clinical practice settings where fractures are treated with vertebral augmentation.
RQ4: What effect does MVA alone, or when compared to KP, have on spinal alignment and fracture risks after VCF?	Adults undergoing MVA procedure alone or when compared to KP for the treatment of OVCF or TVCF	Mechanical augmentation is designed to improve vertebral height restoration and reduce kyphosis, with the intention of reducing associated morbidity.	No geographic, duration of follow up, racial, or gender limitations, all clinical practice settings where fractures are treated with vertebral augmentation.
RQ5: What is the effect of MVA or vertebroplasty or KP on pain and disability outcomes in patients with vertebral compression fractures?	Adults undergoing MVA procedure alone or when compared to KP for the treatment of OVCF or TVCF	Mechanical augmentation may result in better cement distribution and stabilization of fracture which may in turn result in reduced pain and disability.	No geographic, duration of follow up, racial, or gender limitations, all clinical practice settings where fractures are treated with vertebral augmentation.
RQ6: What is the impact of MVA techniques on mortality and morbidity risk in patients with VCF?	Adults undergoing MVA procedure for the treatment of OVCF or TVCF	Multiple large Medicare claims database studies have demonstrated an incremental reduction in 5-year mortality with vertebroplasty and to an even greater extent, KP, compared to non-surgical management of OVCF. However, the potential mortality benefit of MVA, which theoretically offers superior vertebral height restoration and spinal deformity correction, remains inadequately described in the current literature.	No geographic, duration of follow up, racial, or gender limitations, all clinical practice settings where fractures are treated with vertebral augmentation.
RQ7: What are the feasibility and outcomes of treating complex and severe fractures with MVA or KP in those who are poor surgical candidates?	Adults with OVCF or TVCF classified as complete burst fractures, split-pincer fractures, or vertebra plana, who undergo mechanical or KP vertebral augmentation	Anecdotally, mechanical augmentation allows treatment of VCF types that were previously avoided or viewed as having a poor risk/benefit ratio for treatment with traditional vertebral augmentation.	No geographic, duration of follow up, racial, or gender limitations, all clinical practice settings where fractures are treated with vertebral augmentation.

### 2.1 Eligibility criteria

Inclusion criteria required that studies: (1) were peer-reviewed full text journal articles, (2) published in English, and (3) reported at least one relevant clinical, functional, radiographic, or complication-related outcome aligned with one or more of the defined research questions. No restrictions were placed on publication dates. A broad range of study designs was considered, including quantitative, qualitative, and mixed-methods research, to capture diverse perspectives and evidence types. Studies were excluded if they did not investigate MVA as a primary intervention, or if they lacked relevance to the scope of the research questions. Mechanical vertebral augmentation (MVA) was defined a priori as any vertebral augmentation technique employing an expandable intravertebral implant to restore vertebral body height and facilitate cement delivery, excluding traditional balloon kyphoplasty and vertebroplasty. No restrictions were placed on patient age, sex, or geographic location. No protocol for this review was registered or published in advance, and Institutional Review Board approval was not required as this work synthesized previously published literature without involving human participants or identifiable patient data.

### 2.2 Information sources

A comprehensive literature search was conducted using three electronic databases: Ovid Medline, Embase, and Web of Science. The search was executed on September 12, 2023.

### 2.3 Search strategy

The search strategy was developed in collaboration with a medical librarian to ensure rigorous and systematic retrieval of relevant studies. The strategy combined standardized subject headings with keywords and included the following terms and phrases. Search terms were combined using Boolean operators as follows: “compression fracture” “osteoporosis” OR “pathologic decalcification” OR “traumatic” OR “microfracture” OR “spinal burst fracture” OR “vertebra plana”, “vertebroplasty” OR “vertebral augmentation” OR “mechanical augmentation” OR “balloon augmentation” OR “kyphoplasty” OR “bone cement”

“pain” OR “visual analog scale” OR “height” OR “survival” OR “mortality” OR “life expectancy” OR “sagittal balance” OR “segmental kyphosis” OR “disability” OR “functional improvement” OR “spinal alignment” OR “load distribution” OR “mechanical stress” OR “anterior column load” OR “recompression” OR “extravasation” OR “cement leak” OR “adjacent segment fracture” OR “index fracture collapse”.

### 2.4 Selection of sources of evidence

A total of 5680 citations were imported into Covidence, an electronic platform for managing systematic reviews. 2859 duplicate records were identified and removed, resulting in 2821 unique citations [12,13]. Eight reviewers working in pairs evaluated the titles, abstracts, and then full text of all publications identified by our searches for potential inclusion. Disagreements on study selection and data extraction were resolved by consensus and discussion with other reviewers if needed [11].

### 2.5 Data charting process

Data were charted from the selected studies using standardized charting forms in Covidence that had been tested and calibrated by the review team prior to use. Charting was performed independently by two reviewers to minimize bias and ensure consistency. Any discrepancies between reviewers were resolved through discussion between the two reviewers or further adjudication by a third reviewer [11].

### 2.6 Data items

The review aimed to extract comprehensive data from studies reporting outcomes related to compression fractures, with a focus on variables such as study design, sample size, number and anatomical location of spinal levels treated, population characteristics, fracture etiology, fracture classification according to the AO Spine system, and intervention details including MVA device, vertebroplasty, or KP. Key outcome measures included pain scores (assessed via the Visual Analog Scale [VAS] or Numeric Rating Scale [NRS]), functional outcomes (*e.g.*, Oswestry Disability Index [ODI], EQ-5D, EQ-VAS), vertebral height or angular restoration, survival, mortality, and complications, such as cement leakage, adjacent or non-adjacent segment fractures, and reoperation rates. Follow-up duration was also documented. Where outcome definitions were not explicitly provided, standardized definitions were applied to maintain consistency across studies. Outcome data were extracted at the longest reported follow-up within each study. Pain, functional scores, and radiographic alignment outcomes were typically reported between 3 and 12 months. Adjacent vertebral fracture outcomes were extracted at the final reported follow-up (commonly 6–12 months). Cement leakage was classified as an immediate peri-procedural outcome. For the purposes of this review, long-term follow-up was defined as outcomes reported beyond 12 months.

### 2.7 Synthesis of results

Extracted data were synthesized descriptively. Studies were organized and summarized according to the relevant research questions. Data synthesis involved both tabulation and narrative approaches according to predefined research questions. Because of substantial methodological, clinical, and statistical heterogeneity across studies, including variability in device type, fracture morphology, outcome definitions, and follow-up periods, we did not perform quantitative pooling of effect sizes. Instead, when multiple studies reported conceptually similar outcomes (*e.g.*, cement extravasation, adjacent fracture rates, pain or disability scores), we generated forest plots displaying individual study estimates to illustrate the range, distribution [14], and direction of effects across the evidence base without calculating pooled summary estimates. For each outcome, we extracted the effect measure reported in the original study (*e.g.*, incidence or prevalence proportions, or mean change scores with standard deviations for patient-reported outcomes). When sufficient information was available, we calculated study-level estimates and 95% confidence intervals (CIs) to allow consistent visualization across forest plots. These plots were used solely as descriptive tools to enhance interpretability and highlight patterns in the data, rather than for meta-analytic inference. Given the heterogeneity in mechanical vertebral augmentation (MVA) technologies and limited numbers of studies for most devices, we examined all MVA systems together only for visualization purposes, while also presenting device-specific study estimates when available (*e.g.*, SpineJack®, Vertebral Body Stenting, Kiva®). All analyses were performed using Stata/MP 19.0 (StataCorp LLC, College Station, TX).

## 3 Results

**Fig. 2:**
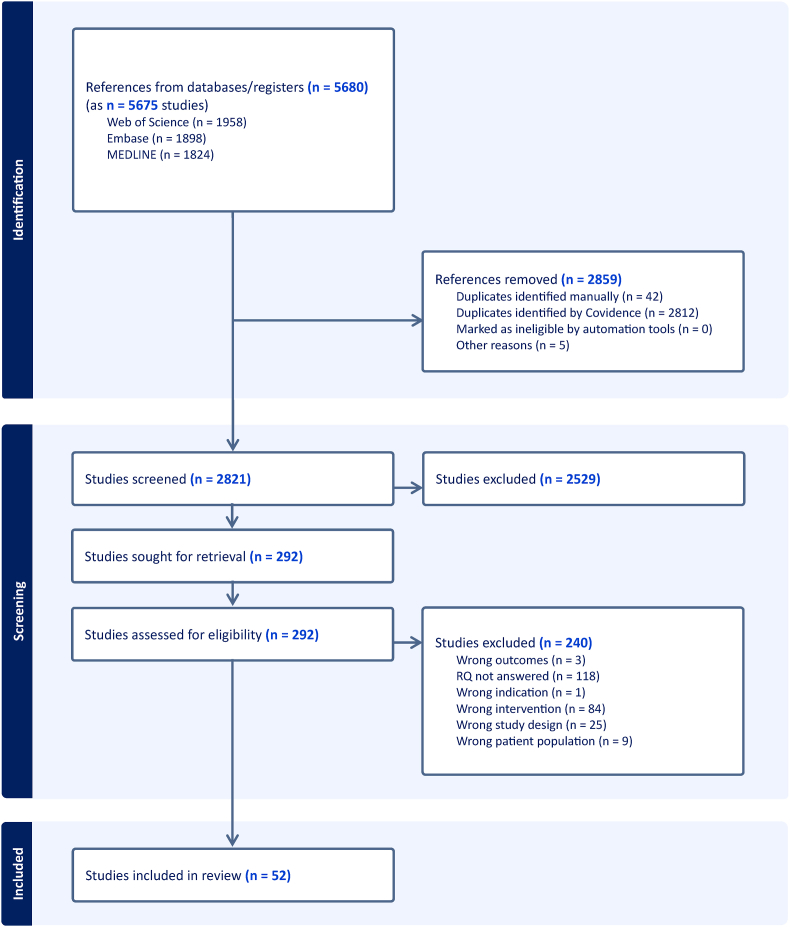
PRISMA flow diagram of study selection.

A total of 5680 records were identified through database searches (Web of Science, Embase, and MEDLINE). After removing 2859 duplicates, 2821 studies were screened, with 292 full-text articles assessed for eligibility. Ultimately, 52 studies met inclusion criteria and were included in the final review and analysis. The study selection process is presented in the PRISMA flow diagram (Fig. 2), and characteristics of the included studies are summarized in Table 2. No studies of vertebroplasty meeting inclusion criteria were found. Results are presented according to the predefined research questions. Because of substantial heterogeneity in data and study design, intervention type, outcome definitions, and reporting methods, no quantitative pooling of data was performed. When multiple studies reported similar outcomes, forest plots were used to display individual study estimates descriptively, and all findings were synthesized narratively.

RQ1: What are the clinically significant ramifications of cement extravasation into adjacent segment discs during MVA procedures, in isolation or when directly compared to vertebroplasty or KP?

**Table 2:** Characteristics of 52 included studies.

Study	Design	Patient Sample	Intervention(s)	Comparator	Clinical (C) and Radiographic (R) Outcome Measures	Follow-up	Cement Leakage/New Fractures
Marie-Hardy 2022 [47]	Retrospective cohort study	53 patients with non-osteoporotic VCF (AOSpine A) at T7-L5	MVA (Tektona®) ± percutaneous fixation	NA	C: NS R: AVH, MVH, PVH, KA	<3, 17 months (avg)	Asymptomatic cement leakage
Yeh 2021 [32]	Retrospective cohort study	354 patients with acute, subacute, or nonunion VCF at T6-L4	MVA (SpineJack®): 60	VP: 88 KP: 124 KP w/IVEP: 46 KP w/vesselplasty: 36	C: VAS R: KA, AVH, PVH	12 months	Symptomatic and asymptomatic cement leakage, new adjacent level fracture
Vendeuvre 2021 [16]	Retrospective cohort study; modeling study	48 patients with non-osteoporotic VCF (Magerl A1, A2, A3) at T12-L2	MVA (VBS): 24	KP: 24	C: NS R: AVH, PVH, KA	1, 90 days	Asymptomatic cement leakage
Chiang 2021 [58]	Retrospective cohort study	62 patients (69 vertebrae) with OVCF	MVA (SpineJack®): 34 (38 vertebrae)	KP: 28 (31 vertebrae)	C: VAS, ODI R: AVH, MVH, PVH, KA	1 week; 12 months	NS
Meyblum 2020 [24]	Retrospective cohort study	51 patients with OVCF (AOSpine A3, A4) at T12-L4	MVA (SpineJack®)	NA	C: VAS R: KA	4-6 weeks; 12 months	Asymptomatic cement leakage, new adjacent level fracture
Chi 2020 [34]	Retrospective cohort study	57 patients with OVCF-associated spinal canal encroachment at T7-L5	MVA (SpineJack®): 16	VP: 41	C: VAS, ODI, EQ-5D R: AVH, MVH, PVH, KA	1 week; 3, 6, 12 months	Cement leakage, new adjacent and nonadjacent level fracture
Noriega 2019a [8]	RCT	141 patients (155 vertebrae) with OVCF at T7-L4	MVA (SpineJack®): 68 (75 vertebrae)	KP: 73 (80 vertebrae)	C: VAS, ODI, EQ-5D, EQ-VAS R: MVH, KA	5 days; 1, 6, 12 months	Asymptomatic cement leakage, new adjacent and nonadjacent level fracture
Arabmotlagh 2019 [22]	Prospective cohort study	31 patients (31 vertebrae) with OVCF (AOSpine A1) at T6-L5	MVA (SpineJack®)	NA	C: VAS R: KA, AVH	3, 12 months	Asymptomatic cement leakage, new adjacent level fracture
Beall 2017 [59]	RCT (secondary safety analysis)	253 patients (177 vertebrae) with OVCF (A1.1, A1.2, A1.3)	MVA (Kiva®): 127 (156 vertebrae)	KP: 126 (155 vertebrae)	C: NA R: NA	30, 90 days; 12 months	New fracture
Lin 2016 [60]	Retrospective cohort study	75 patients with OVCF at T8-L5	MVA (SpineJack®): 36	VP: 39	C: VAS R: KA, AVH, MVH	1 week; 3, 6, 12 months	New adjacent and nonadjacent level fracture
Fan 2016 [40]	Retrospective cohort study	218 patients (236 vertebrae) with OVCF at T10-L5	MVA (Jack vertebral dilator)	NA	C: VAS, ODI R: AVH, MVH, PVH, KA	1 week; 14 months (avg)	Asymptomatic cement leakage, new adjacent level fracture
Ee 2015 [30]	Retrospective cohort study	317 patients with OVCF at T1-L5	MVA (Sky bone expander): 56	VP: 148 KP: 97 CMM: 62	C: VAS, ODI, SF-36 R: KA, AVH	1, 6, 24 months	Asymptomatic cement leakage, new adjacent level fracture
Baeesa 2015 [48]	Prospective cohort study	27 patients with VCF (Magerl A1.2, A1.3, A3.1) at T10-L4	MVA (SpineJack®)	NA	C: VAS R: AVH, MVH, PVH, KA	3, 6, 12 months	Asymptomatic cement leakage, new adjacent level fracture
Beall 2015 [59]	RCT (secondary economic analysis)	253 patients (177 vertebrae) with OVCF (A 1.1, A 1.2, A 1.3)	MVA (Kiva®)	NA	C: NA R: NA	NA	NA
Tutton 2015 [28]	RCT	285 patients with OVCF (A 1.1, A 1.2, A 1.3) at T1-L5	MVA (Kiva®): 144	KP: 147	C: VAS, ODI R: NS	1 week; 1, 6, 12 months	Cement leakage, new adjacent level fracture
Anselmetti 2014 [23]	Retrospective cohort study	40 patients (42 vertebrae) with osteoporotic or traumatic VCF (Magerl A1.1, A1.2, A1.3) at T11-L5	MVA (VerteLift™)	NA	C: VAS, ODI R: AVH, MVH, PVH	24 h; 15 months (avg)	Asymptomatic cement leakage, new adjacent level fracture
Li 2014 [18]	Retrospective cohort study	16 patients with OVCF at T11-L2	MVA (Jack vertebral dilator)	NA	C: VAS R: AVH, MVH, KA	1 day; 1 week; 19 months (avg)	Asymptomatic cement leakage
Eschler 2014 [62]	Prospective pilot study	4 patients with VCF (Müller A1.3) at L1-L4	MVA (OsseoFix®)	NA	C: VAS, ODI, RMDQ R: AVH, MVH, PVH, KA	1, 3 days; 6, 28 months (avg)	No cases of new fracture
Thaler 2013 [19]	Retrospective cohort study	56 patients with OVCF (AO A1)	MVA (VBS): 27 (55 vertebrae)	VB: 29 (61 vertebrae)	C: NS R: AVH, MVH, PVH, KA	3 months (avg)	Asymptomatic cement leakage, new adjacent level fracture
Werner 2013 [17]	RCT	65 patients (100 vertebrae) with OVCF (A1.1, A1.2, A1.3, A3.1)	MVA (VBS): 50 vertebrae	KP: 50 vertebrae	C: NS R: KA	NS	Cement leakage
Korovessis 2013 [15]	RCT	168 patients (255 vertebrae) with OVCF (Genant and Jergas grade ≥1)	MVA (Kiva®): 82 (133 vertebrae)	KP: 86 (122 vertebrae)	C: VAS, ODI, SF-36 R: AVH, MVH, PVH, KA	14 months (avg)	Symptomatic cement leakage, new adjacent and nonadjacent level fracture
Vanni 2012 [31]	Prospective cohort study	300 patients with OVCF (AOSpine A1) at T10-L5	MVA (SpineJack®): 150	KP: 150	C: VAS, ODI R: AVH	1, 6, 12 months	Asymptomatic cement leakage
Hartmann 2012 [64]	Retrospective cohort study	26 patients (29 vertebrae) with traumatic VCF (A3.1, A3.2, A3.3)	KP	NA	C: VAS, ODI, SF-36 R: AVH, PVH, KA	24 h; 15 months (avg)	Asymptomatic cement leakage
Piazzolla 2011 [50]	RCT	50 patients with non-osteoporotic VCF (Magerl A1.2) at T12-L3	MVA (B-Twin® intervertebral spacer): 24 (44 vertebrae)	CMM: 26	C: VAS, ODI R: AVH, MVH, PVH, KA	3, 12 months	Asymptomatic cement leakage
Rosales Olivarez 2011 [43]	Prospective cohort study	57 patients (64 vertebrae) with OVCF at T6-L5	MVA (Kiva®)	NA	C: VAS, ODI R: NS	6 weeks; 3, 12 months	Asymptomatic cement leakage, new adjacent and nonadjacent level fracture
Korovessis 2011 [38]	Prospective cohort study	26 patients (42 vertebrae) with osteoporotic or pathologic VCF at T10-S1	MVA (Kiva®)	NA	C: NRS, ODI R: NS	2, 6 months	Cement leakage, no cases of new adjacent level fracture
Muto 2011 [53]	Retrospective cohort study	20 patients with osteoporotic and traumatic VCF (Magerl A1) at T11-L4	MVA (VBS)	NA	C: VAS, ODI R: VH	6, 12 months	No cases of new adjacent level fracture
Xiong 2010 [44]	Prospective cohort study	25 patients (27 vertebrae) with OVCF (AO type A) at T6-L3	MVA (Sky bone expander)	NA	C: VAS, ODI, RDQ R: AVH, MVH, PVH, KA	1, 6, 12 months	No cases of cement leakage
Liu 2008 [41]	Prospective cohort study	26 patients (35 vertebrae) with OVCF at T8-L5	MVA (Sky bone expander)	NA	C: VAS R: AVH, MVH, PVH, KA	1 day; 1 week; 1, 3 months	Asymptomatic cement leakage
Foo 2007 [20]	Prospective cohort study	40 patients with OVCF (AOSpine A1.2, A1.3) at T12-L1	MVA (Sky bone expander)	NA	C: VAS, NASS-LS, SF-36 R: AVH, MVH, PVH, KA	1 day; 12 months	Cement leakage, new adjacent and nonadjacent level fracture
Zheng 2007 [25]	Prospective cohort study	25 patients (30 vertebrae) with osteoporotic, spinal metastatic tumor, vertebral angioma, and traumatic VCF at T7-L5	MVA (Sky bone expander)	NA	C: VAS, ODI R: AVH, MVH, PVH, KA	14 months (avg)	Symptomatic and asymptomatic cement leakage
Stoffel 2007 [46]	Prospective cohort study	74 patients (118 vertebrae) with OVCF	KP	NA	C: VAS, KPS, SF-36, KPS R: KA	Immediately post-op; 6 weeks; 3, 6, 12, 24 months	Symptomatic and asymptomatic cement leakage, new adjacent and nonadjacent level fracture
Tong 2006 [56]	Prospective cohort study	9 patients (12 vertebrae) with OVCF at T12-L5	MVA (Sky bone expander)	NA	C: NS R: NS	1 month	No cases of cement leakage
Wei 2023 [61]	Retrospective cohort study	33 patients (60 vertebrae) with VCF (Magerl A3) secondary to osteoporosis, trauma, or malignancy	MVA (SpineJack®)	NA	C: NRS or 4-point verbal rating scale R: MVH, KA	10 months (avg)	No cases of cement leakage
Gil-Ortiz 2021 [52]	Case report	1 patient (9 vertebrae) with OVCF at T5-L5 secondary to Cushing disease	MVA (SpineJack®)	NA	C: VAS, ODI R: AVH, MVH, PVH, KA	33 months	Cement leakage
England 2021 [39]	Retrospective cohort study	30 pts (53 vertebrae) with osteoporotic, traumatic, or myeloma-associated pathologic VCF at T7-L4	MVA (SpineJack®)	NA	C: NRS R: AVH, MVH, KA	3 months (median)	New adjacent level fracture
Noriega 2016 [21]; 2019b [33]	RCT	30 patients (32 vertebrae) with OVCF at T7 to L3	MVA (SpineJack®): 15 (16 vertebrae)	KP: 15 (17 vertebrae)	C: VAS, ODI, EQ-VAS R: AVH, MVH, PVH, KA	5 days; 1, 3, 6, 12, 36 months	Asymptomatic cement leakage, new adjacent level fracture
Premat 2018 [27]	Prospective cohort study	19 patients with OVCF (Magerl A3) at T11-L2	MVA (SpineJack®)	NA	C: VAS R: AVH, MVH, PVH, KA	1, 6 months	Asymptomatic cement leakage, new adjacent and nonadjacent level fracture
Renaud 2015 [54]	Case series	77 patients (83 vertebrae) with osteoporotic or traumatic VCF (Magerl A1, A1.2, A2, A3.1) at L1-L2	MVA (SpineJack®)	NA	C: VAS R: NS	35 months (avg)	Symptomatic and asymptomatic cement leakage, new adjacent and nonadjacent level fracture
Otten 2013 [29]	Retrospective cohort study	52 patients (68 vertebrae) with OVCF (AO Spine A1.1, A1.2, or A1.3) at T7-L4	MVA (Kiva®): 26	KP: 26	C: VAS, ODI R: AVH, MVH	3, 6 months	Cement leakage, new adjacent and nonadjacent level fracture
Ender 2013 [35]	Prospective cohort study	24 patients (32 vertebrae) with OVCF (AOSpine A1.1-A1.3, A3.1) at T6- L4	MVA (OsseoFix®)	NA	C: VAS, ODI, SWPS R: AVH, PVH, KA	3 days; 12 months	No cases of cement leakage or new adjacent level fracture
Lofrese 2022 [42]	Case series	57 patients with traumatic VCF (AOSpine A2, A3, A4) at T10–L2	MVA (SpineJack®)	NA	C: VAS, ODI, SWPS, modified Rankin Scale, EQ-5D R: AVH, MVH, PVH, KA	1, 6, 12 months	Asymptomatic cement leakage, new adjacent level fracture
Marcia 2021 [26]	Prospective cohort study	30 patients (37 vertebrae) with OVCF (Magerl A1.1, A1.2, A1.3, A2.1) at T6-L5	MVA (Tektona®)	NA	C: VAS, ODI, SF-36 R: AVH, MVH, PVH, KA	1, 6, 12 months	Asymptomatic cement leakage, new adjacent and nonadjacent level fracture
Wan 2020 [45]	Retrospective cohort study	138 patients (206 vertebrae) with OVCF (AOSpine A3, A4) at T10-L1	KP	NA	C: VAS, ODI R: VH, KA	1, 6 months	Cement leakage, new adjacent level fracture
Shen 2010 [36]	Prospective cohort study	110 patients with OVCF at T10-L5	MVA (Jack vertebral dilator)	KP: 55	C: VAS, ODI R: AVH, MVH, KA	13 months (avg)	Asymptomatic cement leakage
Sietsma 2009 [55]	Cadaveric study	8 vertebrae with OVCF (AOSpine A1.2)	MVA (vertebral jack tool)*:* 4 vertebrae	KP: 4 vertebrae	C: NA R: AVH	NA	No cases of cement leakage or new adjacent level fracture
Jacobson 2020 [65]	Case report	1 patient with secondary vertebral collapse after T12 kyphoplasty for OVCF	MVA (SpineJack®)	NA	C: VAS R: KA	NS	No cement leakage or new adjacent level fracture
Chang 2022 [51]	Retrospective cohort study	65 patients with OVCF (Magerl A3.1, A3.2, A3.3) at T9-L4	MVA (SpineJack®)	NA	C: ODI R: AVH, MVH, PVH, KA	6 months	Asymptomatic cement leakage, new adjacent level fracture
Noriega 2015 [37]	Prospective observational study	103 patients with traumatic VCF (Magerl A1, A2, A3, B1, B2, B3) ± osteoporosis at T9-L5	MVA (SpineJack®)	NA	C: VAS, ODI, EQ-VAS R: KA	48 h; 3, 12 months	Asymptomatic cement leakage, new adjacent level fracture
Jhong 2022 [57]	Modeling study	Numerical lumbar model with an incomplete burst fracture at L3	MVA (SpineJack®) ± percutaneous instrumentation	KP ± percutaneous instrumentation	C: NA R: AVH	NA	NA
Scherer 2022 [49]	Retrospective cohort study	25 patients (25 vertebrae) with split or burst-split VCF (AOSpine A2, A4) at T11-L5	MVA (SpineJack®)	NA	C: VAS R: KA	6 months	Asymptomatic cement leakage, no cases of new adjacent level fracture

Abbreviations: AVH = anterior vertebral height; CMM = conservative medical management; EQ-5D = EuroQol Five-Dimensional Questionnaire; IVEP = intravertebral expandable pillar; KA = kyphotic angle; KP = kyphoplasty; KPS = Karnofsky Performance Scale; MVH = middle vertebral height; MVA = mechanical vertebral augmentation; ODI = Oswestry Disability Index; OVCF = osteoporotic vertebral compression fracture; PVH = posterior vertebral height; NA = not applicable; NASS-LS = North American Spine Society Lumbar Spine Outcome Assessment; NRS = Numerical Rating Scale; NS = not specified; RCT = randomized controlled trial; SWPS = Smiley–Webster Pain Scale; VAS = Visual Analog Scale; VBS = vertebral body stenting; VCF = vertebral compression fracture; VH = vertebral height; VP = vertebroplasty.

Fourteen MVA studies directly addressed intradiscal cement leakage; when reported, these events were almost universally described as radiographically apparent but clinically silent [8,[15], [16], [17], [18], [19], [20], [21], [22], [23], [24], [25], [26], [27]].

In 2013, Korovessis et al. conducted a prospective, randomized study comparing percutaneous KP and MVA [15]. Cement extravasation was identified in 12/122 (9.8%) and 4/133 (3.0%) vertebrae in the KP and MVA groups, respectively. An unspecified number of cases in the MVA group were described as intradiscal or paravertebral leakage, without any reported, clinically relevant symptoms. Other comparative studies found no cases of intradiscal leakage in the MVA groups, whereas asymptomatic leakages were observed in 2.0–20.8% of patients who underwent KP [16,17]. In contrast, Noriega et al. reported higher rates of radiographically-identified intradiscal extravasation in both MVA (46.7%) and KP groups (41.0%) from their international, randomized SAKOS trial [8]. All cases were asymptomatic.

In a retrospective study by Li et al. [18], one (6.3%) of the 16 patients treated with MVA experienced cement extravasation into the lower disc and was asymptomatic. Other studies reported similarly low intradiscal extravasation rates with MVA, ranging from 3.6 to 9.7%, without any documentation of clinically significant symptoms [[19], [20], [21], [22], [23]]. Higher rates of approximately 20% were noted in several studies, without clinical significance [[24], [25], [26]].

A prospective study by Premat et al. that evaluated the feasibility of MVA in the treatment of chronic, kyphotic OVCFs noted 36.8% (7/19) of patients presented with an intradiscal leak on CT, without reporting whether any were clinically symptomatic in the immediate postoperative period [27]. Secondary adjacent level fractures (SALFs) occurred in 21% (4/19) of cases between one and nine months post-procedure. Among these four SALF cases, 3/4 were reported to be symptomatic, and 2/4 (50%) were separately noted to have experienced intradiscal cement leak; however, it was not specified whether both intradiscal leaks occurred in symptomatic patients. Subgroup analysis revealed no association between intradiscal leakage and SALFs.

RQ2: What is the incidence of cement extravasation in MVA procedures in isolation or when directly compared to vertebroplasty or KP?

**Fig. 3:**
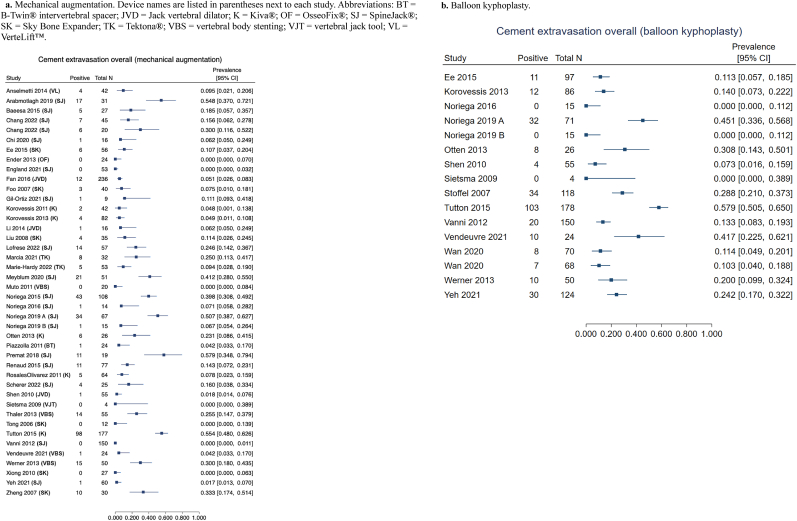
Forest plots of cement extravasation outcomes by treatment group. 3a. Mechanical augmentation. Device names are listed in parentheses next to each study. Abbreviations: BT = B-Twin® intervertebral spacer; JVD = Jack vertebral dilator; K = Kiva®; OF = OsseoFix®; SJ = SpineJack®; SK = Sky Bone Expander; TK = Tektona®; VBS = vertebral body stenting; VJT = vertebral jack tool; VL = VerteLift™. 3b. Balloon kyphoplasty.

A large number of included studies reported cement extravasation rates for MVA, KP, or both, allowing visualization of study-level estimates (Fig. 3a and b). Fewer studies reported extravasation rates for MVA compared to vertebroplasty. Reported rates varied substantially across studies, influenced by differences in device design, cement viscosity, fracture morphology, and whether CT or fluoroscopy was used for detection [8,[15], [16], [17], [18], [19], [20], [21], [22], [23], [24], [25], [26], [27], [28], [29], [30], [31], [32], [33], [34], [35], [36], [37], [38], [39], [40], [41], [42], [43], [44], [45], [46], [47], [48], [49], [50], [51], [52], [53], [54], [55], [56]].

Several comparative studies demonstrated numerically lower leakage rates with MVA versus KP or vertebroplasty. In a randomized trial, Korovessis et al. reported extravasation in 4.9% (4/82) of treated patients in the Kiva® MVA group compared with 14.0% (12/86) in the KP group [15]. Other studies evaluating the Kiva® system reported leakage rates ranging from 23.1 to 55.4%, generally lower than or comparable to KP (30.8–57.9%) within the same cohorts [28,29]. Ee et al. documented a lower incidence of cement extravasation among patients treated with the Sky Bone Expander (10.7%; 6/56) compared to KP (11.3%; 11/97) and vertebroplasty (14.9%; 22/148) [30]. Li et al. observed only one case (6.3%) of intradiscal leakage among 16 patients treated with a jack-based system, and this event was asymptomatic [18]. Reports of vertebral body stenting (VBS) demonstrated variability in leakage rate trends with respect to KP: depending on imaging modality and fracture severity, studies showed leakage frequency with VBS was greater (30.0% vs. 20.0%) or lower (4.2% vs. 41.7%) than KP [16,17]. A single included study comparing VBS to vertebroplasty found lower extravasation rates with MVA (25.5% vs. 41.7%) [19].

Leakage rates for SpineJack® varied across studies but were generally within the lower to mid-range of values reported in the broader MVA literature. Vanni et al. reported no cases among 150 patients treated with SpineJack®, whereas extravasation occurred in 13.3% of patients who underwent KP [31]. The second-lowest incidence of cement extravasation with SpineJack® was 1.7% (compared to 24.2% with KP and 27.3% with vertebroplasty), as reported by Yeh et al. in an observational cohort of 60 patients [32]. In contrast, Noriega et al. saw higher leakage rates with SpineJack® versus KP in both a prospective series at 12 months (50.7% vs. 45.1% of patients) [8] and a randomized trial at 36 months (6.7% vs. 0.0% of patients) [33]. Among 56 patients with OVCF-associated spinal canal encroachment, Chi et al. noted a single case (6.3%; 1/16) of leakage with SpineJack® through the damaged posterior body wall into the spinal canal compared to four instances with vertebroplasty (9.8%; 4/41); none of these events was accompanied by new neurological deficit [34].

When viewed collectively in forest plots, the study-specific estimates of cement extravasation rates show overlapping 95% CI ranges between MVA and KP, albeit with several comparative studies consistently reporting fewer leaks in MVA-treated vertebrae (Fig. 3a and b). Overall, the evidence suggests that cement extravasation occurs with both augmentation techniques, but MVA systems may be associated with lower leakage in certain procedural or fracture contexts. The substantial variation across studies highlights the influence of technique, cement properties, and imaging sensitivity on reported incidence.

RQ3: What effect does structural alignment modification secondary to MVA overall have on outcomes in the setting of VCFs?

Multiple studies evaluated changes in structural alignment following mechanical vertebral augmentation, but only a subset examined whether these radiographic changes were associated with clinical and/or safety outcomes. Five studies specifically assessed the relationship between alignment correction and measures such as pain, function, or secondary adjacent level fracture [22,24,27,35,57].

Jhong et al. performed an analysis of a lumbar finite element model with an L3 incomplete burst fracture, creating seven digital intervention scenarios including complete and incomplete vertebroplasty, percutaneous instrumentation, cement augmentation with SpineJack®, and combinations thereof [57]. All models were compared with an intact lumbar spine. The configuration that most closely approximated normal spinal motion involved cement augmentation combined with SpineJack®. When SpineJack® alone was modeled, lateral bending generated the highest annular ground substance stress. Incomplete vertebroplasty produced marked increases in mechanical loading, with nucleus pulposus stress doubling and annular ground substance stress increasing 2.3-fold relative to the intact spine, along with increased facet joint loading. Based on these findings, the authors concluded that insufficient height restoration may worsen biomechanical stress, whereas implant-mediated restoration may better replicate physiologic conditions.

Ender et al. conducted a prospective, consecutive cohort study evaluating clinical and radiological outcomes of OVCFs treated with the OsseoFix® expandable titanium mesh cage system. At 12-month follow-up, patients showed significant improvements in pain (VAS: 7.7 to 1.4) and disability (ODI: 70.6% to 30.1%). Radiologically, there was a modest but statistically significant improvement in sagittal alignment, with the kyphotic angle according to Cobb decreasing from 11.7° to 10.4°, but no correlation was found between alignment correction and clinical outcomes [35].

In the only study relevant to RQ3 that measured posterior wall protrusion (PWP), Meyblum et al. retrospectively examined outcomes of SpineJack®-treated OVCFs that were considered unstable (per AOSpine classification A3-A4 or with significant PWP >2 mm) [24]. In this cohort of 51 patients without neurological deficits, MVA led to a 34.9% reduction in kyphotic angle and substantial pain relief (VAS: 6.9 to 3.1, *p* < 0.001). Mean preoperative PWP was calculated to be 6.7 ± 2.2 mm (range: 2.5–13.0) and mean postoperative PWP was 6.5 ± 2.2 mm (range: 3.0–13.0; p = 0.02).

Arabmotlagh et al. reported outcomes of SpineJack® in thoracolumbar fractures classified as AOSpine A1, with at least 20% vertebral body height loss or at least 5° of kyphosis [22]. Greater preoperative fracture mobility (defined as the standing-to-supine difference in vertebral height) was significantly associated with greater initial vertebral height restoration and kyphosis correction, as well as greater loss of these corrections at 12 months when initial improvements had partially regressed toward baseline. Pain improvement was not correlated with the amount of initial correction, the degree of correction loss, or the extent of vertebral height restoration over 12 months.

Premat et al. conducted a single-group prospective cohort study evaluating MVA with SpineJack® in 19 patients with OVCFs older than six weeks and kyphotic angles ≥20° [27]. While most patients achieved significant pain relief and kyphosis correction, 21.1% developed SALFs. Compared to patients without subsequent fractures, the SALF subgroup had nearly twice the average kyphosis correction immediately post-procedure (84.5% vs. 45.9%; *p* = 0.007).

RQ4: What effect does MVA alone, or when compared to KP, have on spinal alignment and fracture risks after VCF?

**Fig. 4:**
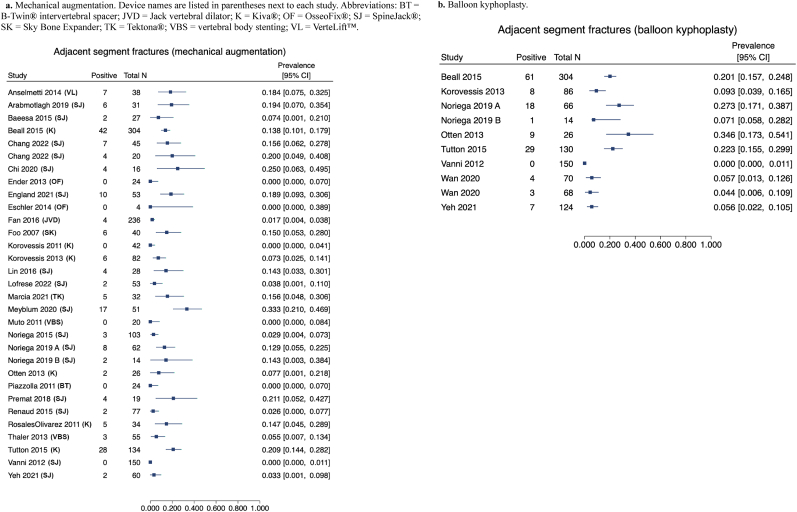
Forest plots of adjacent segment fracture outcomes by treatment group. 4a. Mechanical augmentation. Device names are listed in parentheses next to each study. Abbreviations: BT = B-Twin® intervertebral spacer; JVD = Jack vertebral dilator; K = Kiva®; OF = OsseoFix®; SJ = SpineJack®; SK = Sky Bone Expander; TK = Tektona®; VBS = vertebral body stenting; VL = VerteLift™. 4b. Balloon kyphoplasty.

Multiple studies reported adjacent segment fracture rates following MVA, KP, or both, allowing visualization of study-level estimates in forest plots (Fig. 4a and b). Adjacent fracture rates varied widely across the literature. Across MVA studies, reported adjacent fracture rates ranged from 0% to over 30%, while KP studies demonstrated a similarly wide range. Several comparative studies suggested numerically lower adjacent fracture rates after MVA versus KP [15,28,29,32], although findings differed across devices and study designs. The distribution of estimates in forest plots highlights considerable variability across studies and device platforms without a consistent pattern supporting increased fracture risk with MVA (Fig. 4a) relative to KP (Fig. 4b).

Twelve comparative studies further evaluated alignment correction and subsequent fracture risk between MVA and KP [[15], [16], [17],^21^,[28], [29], [30], [31], [32], [33],^36^,^58^]. These included comparisons of KP with Jack Vertebral Dilator (JVD) [36], Sky Bone Expander (SK) [30], vertebral body stenting (VBS) [16,17], Kiva® [15,28,29], and SpineJack® [21,[31], [32], [33],^58^] in osteoporotic thoracolumbar fractures, using prospective randomized or retrospective cohort designs.

One study comparing JVD with KP found the JVD group demonstrated superior anterior and middle vertebral body height restoration, as well as greater Cobb angle correction; fracture outcomes were not reported [36]. A retrospective study comparing SK with KP reported similar vertebral height and kyphotic correction between groups, but fracture events were pooled across multiple procedures, preventing device-specific interpretation [30]. Two studies comparing VBS with KP found no radiographic differences between groups and did not report fracture outcomes [16,17].

Three studies compared Kiva® with KP [15,28,29], with two reporting no differences in alignment parameters [15,29]. Fracture findings varied: Tutton et al. found no statistically significant differences in secondary adjacent fracture rates (20.9% with Kiva® vs. 22.3% with KP) [28], while both Otten and Korovessis et al. reported fewer fracture events with Kiva® than KP (7.7% vs. 34.6% and 7.3% vs. 9.3%, respectively) without statistical comparison [15,29].

Five studies evaluated SpineJack® in comparison with KP [21,[31], [32], [33],^58^]. Three studies demonstrated greater anterior height restoration with SpineJack® [31,33,58], and two additionally reported greater middle height restoration [33,58]. Two studies reported improved kyphotic correction with SpineJack® [33,58]. Fracture outcomes were inconsistent: one study reported no fractures in either group [31], one noted more patients went on to develop fractures (2 vs. 1) after SpineJack® without statistical analysis [33], and one found no difference between groups despite numerically lower fracture rates with SpineJack® (3.3% vs. 5.6%) [32]. One study did not report subsequent adjacent fracture rates [58].

RQ5: What is the effect of MVA or KP on pain and disability outcomes in patients with VCFs?

**Fig. 5:**
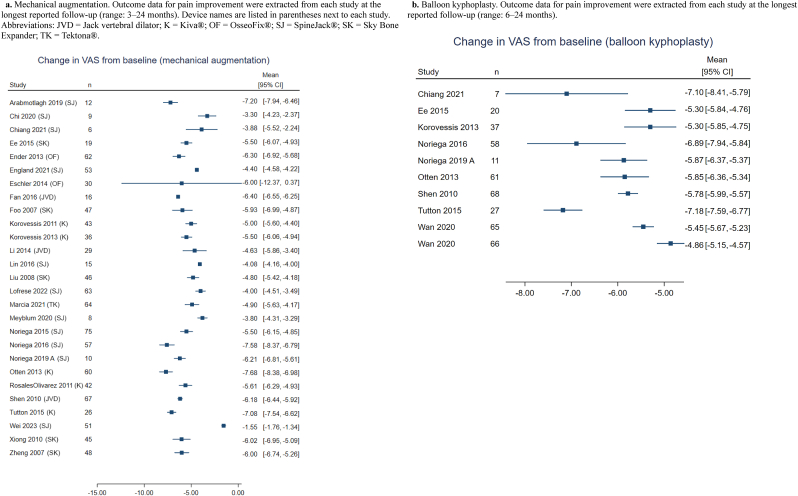
Forest plots of pain improvement (VAS change) by treatment group. 5a. Mechanical augmentation. Outcome data for pain improvement were extracted from each study at the longest reported follow-up (range: 3–24 months). Device names are listed in parentheses next to each study. Abbreviations: JVD = Jack vertebral dilator; K = Kiva®; OF = OsseoFix®; SJ = SpineJack®; SK = Sky Bone Expander; TK = Tektona®. 5b. Balloon kyphoplasty. Outcome data for pain improvement were extracted from each study at the longest reported follow-up (range: 6–24 months).

**Fig. 6:**
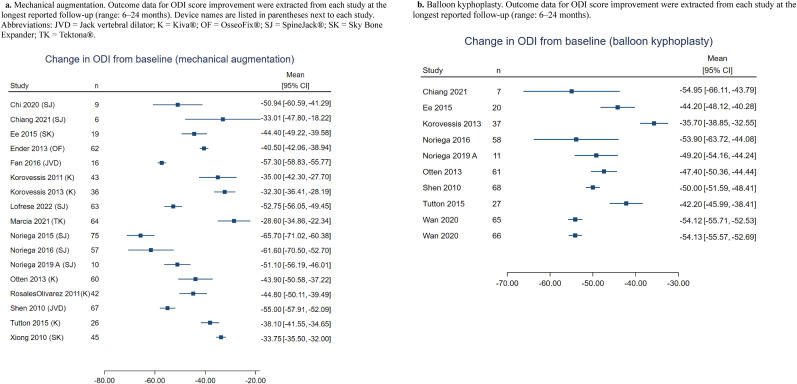
Forest plots of Oswestry Disability Index (ODI) change scores across treatment groups 6a. Mechanical augmentation. Outcome data for ODI score improvement were extracted from each study at the longest reported follow-up (range: 6–24 months). Device names are listed in parentheses next to each study. Abbreviations: JVD = Jack vertebral dilator; K = Kiva®; OF = OsseoFix®; SJ = SpineJack®; SK = Sky Bone Expander; TK = Tektona®. 6b. Balloon kyphoplasty. Outcome data for ODI score improvement were extracted from each study at the longest reported follow-up (range: 6–24 months).

Multiple vertebral augmentation studies reported pain and disability outcomes, which were most commonly assessed using the VAS and ODI [8,[15], [16], [17], [18],[20], [21], [22],[24], [25], [26],[28], [29], [30], [31], [32],[34], [35], [36], [37], [38], [39], [40], [41], [42], [43], [44],[58], [59], [60], [61], [62]]. These outcomes are displayed in forest plots as study-level change scores (final minus baseline), allowing comparison of the magnitude and range of improvement across mechanical augmentation technologies and KP (Fig. 5, Fig. 6b).

### 3.1 Visual analog scale

Across the included studies, MVA consistently showed marked reductions in pain from baseline, with most reporting VAS score improvements of approximately 4 to 7 points within weeks to months after treatment [8,15,18,[20], [21], [22],^25^,^26^,[28], [29], [30],[35], [36], [37], [38], [39], [40], [41], [42], [43], [44],^60^,^62^]. KP studies demonstrated similar decreases in VAS pain scores [8,15,21,[28], [29], [30],^36^,^45^,^58^]. In comparative trials evaluating both MVA and KP within the same cohort, the degree of pain improvement was generally similar between groups, with no consistent evidence indicating superior pain relief with one technique over another [8,15,21,[28], [29], [30],^36^,^58^]. Forest plots demonstrated overlapping distributions of VAS change scores across interventions (Fig. 5a and b).

### 3.2 Oswestry disability index

A similar pattern was observed for disability outcomes. Across MVA studies reporting ODI, patients demonstrated substantial functional improvement following augmentation, with many reporting reductions ranging from approximately 30 to 65 points depending on baseline severity and follow-up duration [8,15,21,26,[28], [29], [30],[34], [35], [36], [37], [38],^40^,[42], [43], [44],^58^]. KP cohorts consistently showed similar improvements, ranging from approximately 35 to 55 points [8,15,21,[28], [29], [30],^36^,^45^,^58^].

Comparative studies likewise showed broadly equivalent ODI improvements between MVA and KP, without a consistent trend favoring one approach [8,15,21,[28], [29], [30],^36^,^45^]. Forest plots of ODI change scores showed substantial overlap between treatment categories, indicating that both techniques yield similar functional benefits (Fig. 6a and b).

RQ6: What is the impact of MVA techniques on mortality and morbidity risk in patients with VCF?

Five studies were included to assess the impact on morbidity and mortality after MVA in the setting of traumatic vertebral compression fractures (TVCFs) or OVCFs. Of the five studies reviewed, only three reported any deaths during the study period [33,37,59]. In all cases, the authors believed that these deaths were unrelated to the procedure itself.

When following patients for three years, Noriega et al. reported one death in the SpineJack® group due to a cardiovascular event approximately 6 months after treatment, and one death in the kyphoplasty group due to a stroke at 21 months [33]. In a separate single-arm cohort from the same author group, two additional deaths occurred: one from renal failure with lower-limb vascular obliteration at two months, and one from acute respiratory failure at seven months [37].

Beall et al. reported a post-hoc analysis of the KAST trial which compared serious adverse events (SAEs) requiring unplanned readmission amongst 300 patients treated with MVA (Kiva®) or KP [59]. While both approaches provided similar clinical efficacy, the MVA group had a 34.4% lower risk of SAE-related readmissions compared to KP. Nineteen deaths were reported at one year, with no significant difference between groups (*p* = 0.849).

Morbidity outcomes beyond cement leakage and subsequent fractures were less consistently reported. Two of the five studies were single-arm and therefore provided limited comparative insight [24,37]. Lin et al. compared SpineJack® with vertebroplasty and found no significant difference in overall complication rates (*p* = 0.57), with similar occurrences of pneumonia and urinary tract infection across groups [60]. In another comparative study, Noriega et al. reported SAEs in five SpineJack®-treated patients and two KP-treated patients; none were attributed to the device or procedure and included events such as bradycardia, cataract, and cholecystitis [33]. The authors of these studies reported that all patients received guideline-concordant osteoporosis management, which may have contributed to the low observed morbidity and mortality rates.

RQ7: What are the feasibility and outcomes of treating complex and severe fractures with MVA or KP/vertebroplasty in those who are poor surgical candidates?

A total of 13 studies analyzing the use of MVA devices in patients with complex or severe fractures who were considered poor candidates for open fusion were included [17,21,24,27,[46], [47], [48], [49],^61^,[63], [64], [65]]. These studies evaluated devices such as intravertebral expandable implants, stent-KP, Tektona®, and/or KP for the treatment of complex or severe thoracolumbar fractures, including AO-Spine A3 burst fractures, AO-Spine A4 burst fractures, split or burst-split fracture patterns, and vertebra plana. Study designs varied and included single-arm prospective and retrospective series, comparative cohorts, and one randomized controlled trial. Across all studies, aspects such as feasibility, radiographic correction, degree of clinical improvement, and complication rates were reported.

### 3.3 Feasibility and safety

All 13 studies included patients with complex vertebral fractures who were not candidates for fusion surgery and reported successful deployment of MVA or KP. No conversions to open surgery were reported. There were uniformly high success rates, and even in cases with canal encroachment, augmentation was performed without new neurological deficits. Cement leakage occurred in a minority of procedures, most frequently into the disc space or paravertebral soft tissues, and was generally asymptomatic [46,48,64]. In one case, MVA was successfully used to augment a failed KP procedure involving inadequate cement fill in a T12 burst fracture [26].

### 3.4 Radiographic outcomes

Across studies, both MVA and KP were associated with meaningful improvements to vertebral alignment and height restoration in acute complex fractures [17,61,[63], [64], [65]]. Mechanical devices like SpineJack®, Tektona®, and vertebral body stents demonstrated greater anterior height restoration compared with KP [17,47,48,63]. Although some loss of correction was observed over time, alignment at follow-up generally remained superior to baseline [17,24,47,48,61,63,64].

### 3.5 Clinical outcomes

Patients consistently experienced rapid improvements in pain and function, often within days to weeks following the procedure, with benefits maintained over short-to medium-term follow-up [17,21,46,64]. Comparative studies and the single randomized trial showed similar levels of pain relief between MVA and KP, though some radiographic parameters favored MVA [17,47,48,63]. No study found immediate or delayed neurological deterioration attributable to the intervention.

### 3.6 Complications

Although varying, cement leakage rates were within the ranges documented for vertebral augmentation in less complicated fracture types. There were no reported cases of symptomatic canal extravasation associated with MVA [46,48,61,64]. The incidence of secondary adjacent-level fractures varied by device and cohort, with higher rates noted in studies where substantial kyphosis correction was achieved, suggesting that aggressive correction may increase adjacent segment loading [17,47,48].

## 4 Discussion

OVCFs can cause significant pain, limit function, decrease quality of life, and even increase mortality over time [66,67]. While vertebral augmentation has evolved into the third iteration of procedure types, conclusions have not been clear or uniform regarding the clinical relevance of cement leakage into adjacent discs, the incidence of cement leaks with MVA, the relationship between structural and clinical outcomes, and the morbidity, mortality, and feasibility of MVA in complex or unstable fractures. Taken together, the available evidence suggests that mechanical vertebral augmentation is a viable and generally safe treatment option and may offer procedural advantages, especially in more complex fracture patterns. However, the current literature is limited by heterogeneous study designs and inconsistent reporting methods. As a result, while MVA demonstrates promise, its comparative clinical benefit over existing augmentation strategies has not yet been clearly established.

One important area explored in the literature is whether cement extravasation into adjacent discs results in meaningful clinical consequences. Across published studies evaluating MVA devices, intradiscal leakage appears uncommon and largely asymptomatic, despite higher imaging-detected rates in some series [8,[15], [16], [17], [18], [19], [20], [21], [22], [23], [24], [25], [26], [27]]. Overall, most studies reporting cement extravasation with MVA did not stratify results by anatomical location. This is important because the clinical implications of leak into the spinal canal versus intradiscal space, for example, are quite different. Reported extravasation rates are further influenced by variability in definitions and imaging modalities across studies. Computed tomography is generally more sensitive than fluoroscopy for detecting small or asymptomatic leaks, which may lead to underestimation in some reports and limit direct clinical comparability. When documented, intradiscal leakage was not always differentiated from paravertebral leakage, limiting interpretability and preventing firm conclusions. Based on currently available evidence, intradiscal cement extravasation associated with MVA does not appear to result in consistent clinically meaningful adverse effects.

Beyond symptom relevance, another important consideration is how frequently cement extravasation occurs across augmentation techniques. Across the studies included in this review, MVA generally demonstrated lower or comparable leakage rates relative to KP, with several randomized or comparative trials reporting notably fewer leaks in certain mechanical implant systems [8,15,17,21,28,31,50]. However, other studies showed similar incidences between techniques or only modest differences, reflecting the influence of device type, cement viscosity and handling, fracture morphology, and procedural technique [8,15,17,21,50]. Because reporting methods, imaging modalities, and definitions of extravasation varied considerably across studies, the apparent trend toward reduced leakage with MVA should be interpreted cautiously.

Another major topic discussed in the literature involves whether structural alignment changes translate into improved clinical outcomes. The limited number of studies assessing both radiographic parameters and patient-reported measures provides mixed and inconclusive findings. Some investigations reported no clear association between improvements in Cobb angle and changes in pain or disability scores [35], while others suggested that specific mechanical devices may influence features such as posterior wall protrusion through ligamentotaxis, although the clinical relevance of these observations remains uncertain [24]. While Kiva®-based MVA has been hypothesized to improve pulmonary function due to sagittal correction, this effect has not yet been demonstrated and remains theoretical [59,[68], [69], [70]].

Emerging evidence suggests a complex relationship between vertebral height restoration and mechanical risk. Some studies have reported that greater correction may be accompanied by increased subsidence or a higher likelihood of secondary adjacent-level fractures, as seen with SpineJack® in the cohorts described by Arabmotlagh and Premat et al., where more substantial kyphotic angle correction appeared to correspond with greater mechanical stress at the treated and adjacent segments [22,27]. Comparative studies evaluating MVA and KP have also shown mixed results: although MVA often achieves greater height restoration and improved alignment, subsequent fracture rates varied across studies, with some reporting fewer adjacent fractures after MVA and others finding no difference or reporting events without statistical comparison [21,[31], [32], [33]]. Overall, while some evidence suggests that greater height restoration may be associated with a reduced risk of subsequent fractures in certain contexts, outcomes also appear to depend on factors such as fracture morphology, the degree and pattern of correction achieved, cement containment, and implant-specific load redistribution. Given the substantial variability in reporting methods, follow-up durations, and device characteristics across studies, the extent to which alignment correction can be optimized to minimize stress transfer and fracture risk remains uncertain.

Pain and functional outcomes improved across all augmentation techniques, with no consistent clinical advantage observed between mechanical augmentation and balloon kyphoplasty. Multiple comparative and single-arm studies demonstrated substantial reductions in pain and disability following both MVA and KP, including cohorts treated with OsseoFix® [26], SpineJack® [22,24,27], and jack-based or balloon-based systems [15,18]. Although mechanical systems frequently achieved greater structural correction, these radiographic differences did not appear to reliably translate into superior improvements in VAS or ODI. The overall pattern of findings suggests that symptomatic relief may be primarily driven by fracture stabilization and reduction of painful micromotion—an interpretation supported by both clinical series and biomechanical modeling, which highlight the role of load redistribution independent of alignment magnitude [57]. Notably, relatively few studies reported outcomes beyond 12 months, limiting interpretation of long-term durability.

Morbidity and mortality outcomes have also been reviewed, although current evidence remains limited. Large population-based analyses suggest that vertebral augmentation may confer a mortality benefit compared with non-surgical management, with this effect reported across multiple countries and persisting over extended follow-up periods [4,71,72]. One Medicare-based study estimated a number needed to treat (NNT) of 11.9 for KP and 23.8 for vertebroplasty at five years [73]. However, other analyses have reported equivocal or absent mortality benefit when compared with conservative management, and variation in findings may reflect differences in patient frailty, comorbid burden, and attribution of cause of death rather than procedural effect [74,75]. To date, no comparable evidence exists describing whether MVA confers similar survival advantages, and among studies reporting mortality events during follow-up, deaths were few and consistently considered unrelated to the procedure [33,37,59]. Limited data on morbidity similarly suggest that serious adverse event rates with MVA are generally low and may be comparable to or lower than those observed with kyphoplasty or vertebroplasty [33,59,60]. Prior work in vertebral augmentation has also proposed physiologic benefits related to height restoration and sagittal realignment, including improvements in pulmonary function [[68], [69], [70]], raising the possibility that devices achieving greater structural correction, such as some MVA systems, could influence respiratory outcomes, although this remains unproven.

Feasibility in more complex fracture patterns has also been investigated. For patients with severe vertebral fractures who are not candidates for open fusion, available evidence suggests that MVA is generally a safe therapeutic option [17,21,24,27,[45], [46], [47], [48], [49],^61^,[63], [64], [65]]. Historically, complex fracture morphologies such as complete burst, split pincer, and vertebra plana have been considered challenging to manage with traditional vertebroplasty or KP due to concerns related to inadequate height restoration, limited cement containment, and risk of neurological compromise from canal or foraminal extravasation. However, MVA techniques, particularly those utilizing expanding intravertebral implants, appear not only safe but potentially advantageous in these settings. Their controlled expansion and ability to restore vertebral body height more predictably may improve deformity correction without increasing the risk of cement leakage or nerve injury [17,24,[46], [47], [48],^61^,^63^,^64^]. In medically frail or high-risk patients, these approaches offer a minimally invasive option for restoring alignment, reducing pain, and facilitating early mobilization. However, longer-term durability and the potential for late structural changes require further study [17,21,[46], [47], [48], [49],^61^,^63^,^64^].

### 4.1 Future research priorities

This scoping review identified several gaps across clinically important domains. Future studies should aim to compare the long-term clinical outcomes after both MVA and KP and correlate these with augmentation durability, subsidence risk, and biomechanical impacts on adjacent segments, particularly in relation to alignment correction. Additionally, comparative trials should evaluate how different MVA devices affect structural outcomes such as sagittal alignment and vertebral body height restoration and determine whether these changes correlate with improvements in pain and function. Studies should stratify high-risk fracture types (*e.g.*, vertebra plana, burst fractures), degree of osteoporosis, and other known risk factors to help clarify which populations will benefit most from MVA.

### 4.2 Limitations

This scoping review excluded non-English publications, which may have contained relevant data addressing the research questions. Only human studies were included, with cadaveric and finite element analyses incorporated exclusively for RQ3, and animal or in-vitro studies omitted. Considerable methodological variability in study designs, reporting standards, and outcome definitions also limited the ability to meaningfully compare findings across studies. As a result, the findings should be interpreted as exploratory rather than definitive. Additionally, this scoping review was not prospectively registered (*e.g.*, PROSPERO), which may limit methodological transparency and introduces the potential for selection or reporting bias.

### 4.3 Clinical takeaways

• MVA is a safe and feasible option for treating VCFs, including complex patterns such as vertebra plana and burst fractures.
• Cement leakage rates may be lower or comparable in MVA relative to KP, but inconsistencies in imaging techniques and definitions across studies limit firm conclusions.
• Structural correction (*e.g.*, height restoration and kyphotic angle improvement) is greater with MVA, yet these improvements do not consistently translate into superior pain or functional outcomes.
• MVA appears particularly advantageous in complex or unstable fractures, where traditional vertebroplasty or KP may be less effective or carry higher risks.

## 5 Conclusion

Current evidence indicates that MVA is a feasible and generally safe treatment option for vertebral compression fractures, including more complex morphologies. MVA may offer technical benefits such as improved height restoration and lower cement extravasation risk compared with KP, although symptomatic extravasation appears rare across all augmentation techniques. High-quality, standardized studies will be essential to clarify how different augmentation strategies perform across diverse fracture types and patient populations, and whether specific structural advantages translate into meaningful clinical benefit.

## Funding

This review was supported by an investigator-initiated research grant from the Skaggs Research Foundation (paid directly to the University of Utah). The sponsor had no role in the design or conduct of the review or in approving the final manuscript. The protocol, search, data extraction, and statistical analysis were developed and performed independently.

## Conflict of interest statement

Zachary L. McCormick, MD serves on the Board of Directors of the International Pain and Spine Intervention Society (IPSIS), has research grants from Avanos Medical, Boston Scientific, Relievant Medsystems, Saol Therapeutics, Spine Biopharma, SPR Therapeutics, Stratus Medical (all paid directly to the University of Utah), and also consultancies with Avanos Medical, Saol Therapeutics, Stryker, and OrthoSon (all relationships ended). Aaron Conger, DO received research grant funding from Stratus LLC (paid directly to the University of Utah).
